# Community interactions and phonemic inventories in emerging sign languages

**DOI:** 10.1017/S0952675721000336

**Published:** 2022-02-21

**Authors:** Diane Brentari, Rabia Ergin, Ann Senghas, Pyeong Whan Cho, Eli Owens, Marie Coppola

**Affiliations:** University of Chicago; Tufts University; Barnard College; University of Michigan; University of Chicago; University of Connecticut

## Abstract

In this work, we address structural, iconic and social dimensions of the emergence of phonological systems in two emerging sign languages. A comparative analysis is conducted of data from a village sign language (Central Taurus Sign Language; CTSL) and a community sign language (Nicaraguan Sign Language; NSL). Both languages are approximately 50 years old, but the sizes and social structures of their respective communities are quite different. We find important differences between the two languages’ handshape inventories. CTSL’s handshape inventory has changed more slowly than NSL’s across the same time period. In addition, while the inventories of the two languages are of similar size, handshape complexity is higher in NSL than in CTSL. This work provides an example of the unique and important perspective that emerging sign languages offer regarding long-standing questions about how phonological systems emerge.

## 1 Introduction

The earliest stages of the emergence of a phonological system should reveal critical aspects of the nature of phonology, in terms of its mechanisms and motivations. In this paper, we ask how phonological systems arise in two different young sign languages. A number of perspectives in previous scholarship have been employed to address how a phonological system naturally emerges, and the current study seeks to add to their insights. Acquisition studies are very informative about changes due to learning and maturational considerations (Newport 1990, Gerken et al. 2011). Studies of iterative learning can carefully control variables such as the number of exposures and the number of members in a communicative interaction (Kirby et al. 2008, Smith et al. 2017, Kocab et al. 2019). Historical and typological studies offer excellent resources for studying language change and variation across long temporal periods and an extensive geographical range (Blevins 2004, Bybee 2011, Maddieson et al. 2011, Trudgill 2011, Dryer & Haspelmath 2013, Mielke 2013). These approaches do not, however, capture the precise moment in historical time at which articulatorily complex phonological units are evident, but before there is a mature phonological system in place.

The present study captures this moment by examining two naturally emerging languages in the earliest stages of development. The languages are the first languages of the signers in our study, so there is no possible interference from a preexisting language. Our data come from two types of emerging sign languages, in order to observe the effects on a phonological inventory of community size and type of contact among members (Meir et al. 2010, Meir et al. 2012). In village sign languages, transmission of the language takes place within extended families. Both deaf and hearing family members play a role as linguistic models and as acquirers, and many children, both hearing and deaf, acquire the language from birth, or very early in life. In COMMUNITY SIGN LANGUAGES, transmission occurs at school or other places where deaf people gather. Signers of community sign languages typically have varied backgrounds, and there are very few hearing people in the community. One of the sign languages in the current study is a village sign language used in Turkey (Central Taurus Sign Language; CTSL), and one is a community sign language (Nicaraguan Sign Language; NSL). We discuss the details of these two communities in §2.1.^1^

The creation of a sign language does not begin with people who set out to create an organised system; it begins with deaf individuals who seek to communicate. Initially, deaf people use gestures to communicate with the hearing people around them, devising a method for communication with family and friends which in time becomes systematic, and which is the primary means of communication for the deaf individual. A key ingredient needed for a sign language grammar to emerge is therefore thought to be the system’s use as a primary communication system (Brentari & Coppola 2013). Homesigners have this ingredient, being deaf individuals who use an idiosyncratic system that they themselves created. Previous work has demonstrated that patterned, linguistic behaviour is lacking in the co-speech or silent gestures of the hearing people who provide input to the homesigner (Goldin-Meadow 2003, Hunsicker & Goldin-Meadow 2012, Flaherty et al. 2021). Carrigan & Coppola (2017) discuss limitations in comprehension of homesigners’ systems by hearing family members, including those in Nicaragua who are the target of this study, particularly the lack of fluent two-way communication which involves producing the system and seeing the system produced by others at the same level of fluency. Because no one in their family has learned a preexisting sign language, or uses the homesign system as fluently, homesigners are linguistically isolated.

Horizontal contact occurs when homesigners come together for the first time and communicate regularly with one another. Subsequently, members of the community who have had horizontal contact become the language models for the next cohort or generation of users. These subsequent cohorts thereby experience what we refer to as vertical contact with proficient language models in their environments, beyond home-signing peers (see §2 for more details). Horizontal and vertical contact are two factors shown to be important in developing grammar on many levels (Senghas & Coppola 2001, Senghas 2003, 2005, Senghas et al. 2005, Gagne 2017). For example, in Nicaragua, more stable lexical forms have been found in signers who have had horizontal contact than in home signers (Richie et al. 2014), and even more stability is found in signers who have had vertical contact than those with only horizontal contact (Goldin-Meadow et al. 2015). Spatial modulations also become more broadly and consistently applied (Senghas & Coppola 2001), and the use of points expands from locative to nominal functions across cohorts (Coppola & Senghas 2010). Similar differentiation in grammatical complexity and lexical stability across cohorts has been found in Al-Sayyid Bedouin Sign Language (ABSL), a village sign language, and in Israeli Sign Language, a community sign language (Sandler et al. 2005, Meir et al. 2010, 2012, Padden et al. 2010, Israel & Sandler 2011, Sandler, Aronoff et al. 2011).

We use a model of first-language acquisition as an important anchor for our work for a number of reasons. First, in the current study, the sign language in question is the deaf signers’ first language. Second, more rapid change in NSL between cohorts suggests that younger children may have more language-creating abilities than older children, or at least more years to apply such abilities. Early exposure typically enables better language learning (Newport 1990), and previous research has demonstrated that children’s advantage in language acquisition also allows them to create linguistic structures when the input is sparse or inconsistent (Singleton & Newport 2004). A regular influx of new deaf signers into the NSL community may lead to more rapid change in a community sign language relative to a village sign language (Senghas 2005). Third, the group of signers who are the most systematic in their use of innovation are those with vertical contact who also have the advantage of entering the system at a young age (typically before age 7), suggesting that language change is fuelled in part by first-language acquisition (Senghas & Coppola 2001, Senghas et al. 2004).

In a village sign language, new signers are added to the community slowly, restricted by the number of deaf infants who are born into the community. The slow addition of new deaf, first-language users may in turn slow the rate of change in CTSL. Moreover, a high degree of familiarity among community members may allow signers to take advantage of shared knowledge, thus requiring a village sign language to be less linguistically explicit than the languages used by communities whose members do not have as much in common (Meir et al. 2010, 2012).

The object of analysis in this study is handshape, a well-studied component of sign language phonology (Fenlon et al. 2018, Brentari 2019 and references therein). It has been 60 years since it was first determined that sign languages have a phonological level of grammar (Stokoe 1960), and a great deal of work on sign language phonology since then has shown that phonological analyses of a new communication modality can shed light on old questions. In the following section we describe the key findings of this work, and how we use it to address phonological emergence.

We go beyond previous work on phonological emergence in the following ways. First, we analyse how phonological structure varies and changes across two languages and across cohorts, comparing the size and complexity of their handshape inventories, as well as considering the role of learning biases. Second, we measure the effect of a set of non-linguistic factors, specifically community size and internal social dynamics, on the size and complexity of phonological inventories. By closely comparing CTSL and NSL, we suggest how some factors might be prioritised over others in a phonological system that is developing over time and in the context of different social factors.

### 1.1 Phonological factors

**Figure 1:**
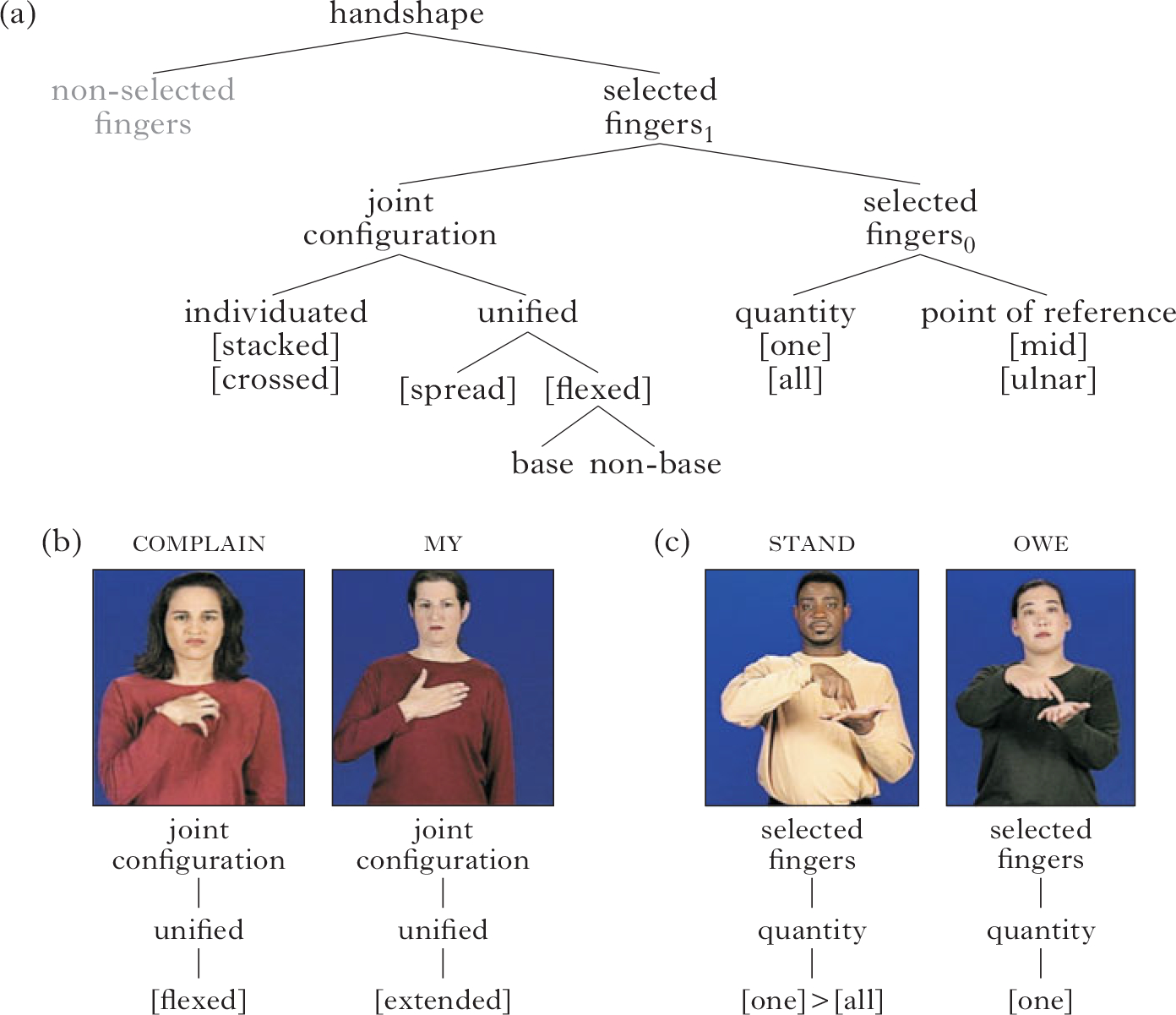
(a) Phonological representation for handshape in the Prosodic Model (Brentari 1998, 2019), along with ASL minimal pairs for (b) joint configuration (complain, my) and (c) selected fingers (stand, owe). The two-fingered handshape in stand is represented by the features [one] and [all] being in a dependency relation ([one]>[all]); see van der Hulst & van der Kooij (2021). The structure does not show features that would be filled in by default, e.g. uncrossed, unstacked, unflexed and radial point of reference.

Previous work has demonstrated that there are five sign languages parameters which have phonological status – handshape, movement, place of articulation, orientation and non-manual behaviours (Stokoe 1960, Stokoe et al. 1965, Klima & Bellugi 1979, Liddell & Johnson 1989). For a number of reasons, we focus on the handshape parameter. First, there is relatively strong consensus about its phonemic status, its autosegmental status and its hierarchical structure (Sandler 1989, van der Hulst 1993, Brentari 1998, van der Kooij 2002, Sandler & Lillo-Martin 2006). The properties relevant for this analysis are expressed in the hierarchical structure shown in Fig. 1a for the dominant hand (the hand used in producing one-handed signs), as formulated in the Prosodic Model of sign language phonology (Brentari 1998, 2019). All of the branches of structure in Fig. 1a meet at least one of the criteria set forth in Clements (2001, 2003) for features. The feature may be responsible for a minimal pair, be used in a phonological rule or have morphological status. The structure in Fig. 1a highlights the two sets of features in this analysis – joint configuration (the postures of the finger joints) and selected fingers (those fingers that can touch the place of articulation, and which can change during the course of a sign’s production). Joint configuration and selected fingers are sister nodes in the hierarchical structure of at least four models of sign language phonology (Sandler 1989, van der Hulst 1993, Brentari 1998, 2019, van der Kooij 2002).

A second reason for handshape being a good choice as an object of study is that it exhibits minimal pairs specifically for joint configuration and selected finger features in many sign languages (Sandler 1989, Brentari 1998, 2019). For example, ASL complain 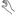 *vs*. my 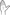 (Fig. 1b) is a minimal pair based on joint configuration features, and ASL stand 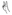 *vs*. owe 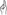 (Fig. 1c) is a minimal pair based on selected finger features.

Third, selected finger and joint configuration features have independent distributions within the handshape systems of several well-established sign languages (Friedman 1976, Mandel 1981). According to the Selected Finger constraint (Sandler 1989), selected finger features appear only once per sign, while joint features can change within a sign as the hand opens and closes.

Based on previous literature on emerging phonological systems, we take as given that minimal pairs and rules, such as the Selected Finger constraint, are not robust in very young sign languages (Sandler, Aronoff et al. 2011, Brentari et al. 2012, Coppola & Brentari 2014). Thus far, we have not found minimal pairs or phonological rules in CTSL or NSL. Van der Kooij & van der Hulst (2005) also point out that there are few minimal pairs even in well-established sign languages. We therefore introduce a new method for including a feature in an inventory, which is based on near-minimal pairs instead of minimal pairs (see §3.1).

Joint configuration and selected finger features can be assigned complexity scores based on their structural complexity (Brentari et al. 2012, Aristodemo 2014), frequency (Hara 2003, Eccarius & Brentari 2007, Caselli & Pyers 2017) and age of acquisition in first language acquisition (Boyes Braem 1990, Marentette & Mayberry 2000, Morgan et al. 2007). For example, fully open 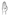 and fully closed 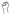 joint configurations and selected finger groups with all of the fingers 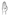 and the index finger 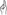 are acquired at the first stage of handshape acquisition, at approximately 24 months. They are also the most frequent joint configurations and selected finger groups across many sign languages (Hara 2003), and have the simplest structures in the phonological representation. They are also argued to be easier to produce (Ann 2006) and to perceive (they are less confusable; Lane et al. 1976) than other handshapes. These criteria can be applied in like manner to medium- and high-complexity features, such as [stacked] and [crossed]. In many sign languages, handshape inventories are skewed toward low-complexity handshapes. In ASL 89% of handshapes have low selected finger complexity, and 74% have low joint configuration complexity (Hara 2003, Eccarius & Brentari 2007, Eccarius 2008).

**Figure 2:**
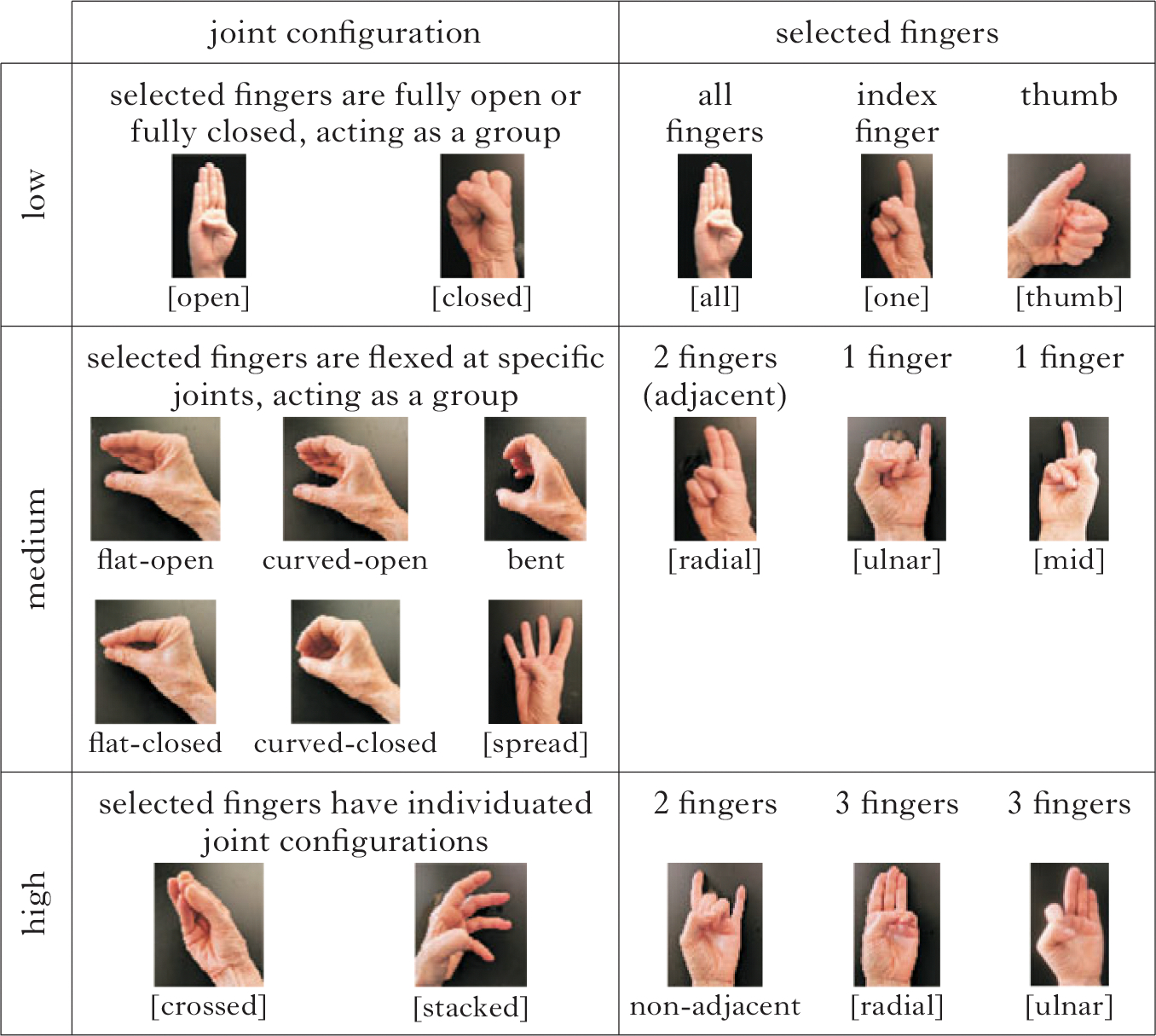
Low-, medium- and high-complexity representative handshapes for features along the dimensions of joint configuration (left) and selected fingers (right). The handshapes varying in joint complexity levels (left) are illustrated with the B-handshape group (the whole hand), and handshapes varying in selected finger complexity levels (bottom) are illustrated with fully extended fingers.

Using the criteria of (i) frequency, (ii) age of acquisition and (iii) phonological structure, we can assign three levels of complexity (1, 2, 3) to joint configuration and selected finger features, as illustrated in Fig. 2 and described in (1). The handshapes in Fig. 2 are examples of how the complexity levels align with the features and structures in Fig. 1.^2^

1. *Complexity defined by feature group*
  - *Joint configuration*
    - Low
      The finger joints act together as one unit. All finger joints are fully open or fully closed.
    - Medium
      The finger joints act together as one unit. Specific finger joints are flexed, either the metacarpophalangeal joint or the proximal interphalangeal joint.
    - High
      The fingers act independently (not as a unit), either by crossing each other or stacking on top of one another.
  - *Selected fingers*
    - Low
      The thumb, the whole hand or the index finger. The index finger is represented as a [one] feature with the default, radial point of reference.
    - Medium
      Two adjacent fingers with the default, radial point of reference, or a single finger with a marked point of reference: [mid] or [ulnar].
    - High
      Three-finger handshapes, or two-finger handshapes with a marked point of reference.

Joint configuration and selected finger features not only create phonemic contrasts, as in Fig. 1; these two groups of features are also associated with two iconic, meaningful handshape classes in the morphological system of many sign languages: object handshapes, which represent the shape of objects, and handling handshapes, which represent how objects are manipulated or used (Supalla 1982, Emmorey 2003). Object and handling handshapes also show a morphosyntactic opposition (Zwitserlood 2003, Benedicto & Brentari 2004). Object handshapes are associated with no-agent clauses (typically intransitive), and handling handshapes are associated with agentive clauses (typically transitives). These distributional patterns of object and handling handshape classes are found in both NSL (Goldin-Meadow et al. 2015) and CTSL (Ergin & Brentari 2017). A handshape class distinction for meaning does not, however, speak to how the two handshape classes are expressed phonologically.

**Figure 3:**
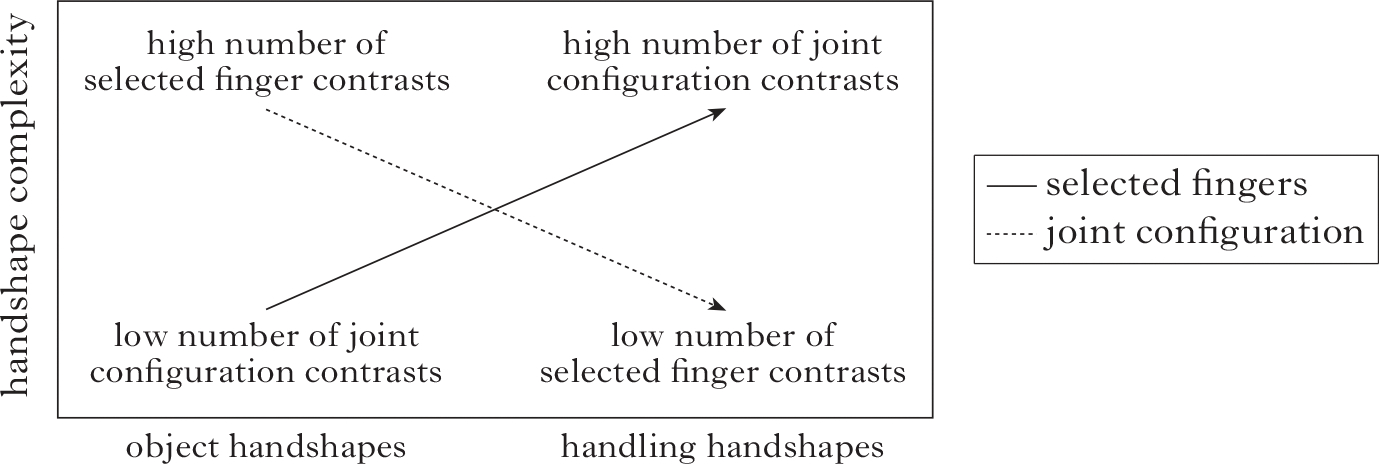
The patterns of joint configuration and selected finger features in (left) object handshapes and (right) handling handshapes in well-established sign languages.

In various sign languages, a double dissociation in phonological form has been reported in the use of joint configuration and selected fingers in handling and object handshape classes (Brentari & Eccarius 2010, Brentari et al. 2012, Brentari et al. 2017). We observe low joint complexity and high selected finger complexity in object handshapes (Fig. 3, left), and high joint complexity and low selected finger complexity in handling handshapes (right). This pattern does not appear in silent gesture, and it is only partially evident in homesigners (Brentari et al. 2012, Coppola & Brentari 2014).

The current study is the first analysis of dynamic changes across groups in emerging sign languages and adult homesign systems, and we want to determine the groups for which this double dissociation holds. We predict that the deployment of joint configuration and selected finger features will not happen in a uniform fashion during the emergence of phonology, but instead be affected by the class of handshape (object, handling), by learning bias and by non-linguistic factors, discussed in the following sections.

We are working with young sign languages in which there is a great deal of variation in the use of a ‘core’ lexicon (Israel & Sandler 2011, Richie et al. 2014, Ergin et al. 2021). To ensure we are analysing comparable forms across cohorts and languages, we therefore target handshapes whose meanings are iconic. We believe our findings will be valid not only for these iconic handshape classes (handling, object), but for the entire handshape inventory, because Eccarius (2008) found a high correlation between the complexity scores of iconic, classifier handshapes and handshapes in the core lexicon. In a study of three unrelated sign languages, American Sign Language (ASL), Swiss German Sign Language (DSGS) and Hong Kong Sign Language (HKSL), Eccarius found that the ordered ranking of handshape complexity for the three sign languages was the same for both core vocabulary and classifier constructions: HKSL > ASL > DSGS.

### 1.2 Formal and substantive bias

Formal bias and substantive bias have been discussed in the spoken language literature as possible motivations for a phonological system to grow or change in specific ways. Moreton & Pater (2012a, b) describe these two types of bias, and suggest that, in spoken language, phonetic effects (a type of substantive bias) are less reliable than the effects of abstract structural complexity (formal bias). Here we test a version of that claim with data from the two emerging sign languages; we expect that both structural and substantive bias are motivating factors in an emerging handshape inventory.

Formal (structural) bias refers to the number of abstract units in a particular structure within a given system; the higher the number of structures involved in a form’s representation (i.e. features, syllables, segments, etc.), the more formally complex it is. Thus, formal bias refers to the tendency for simpler formal structures to be easier to learn and to be more diffuse cross-linguistically than more complex ones. For example, in a spoken language consonant system, the set of glottal features is more formally complex if voicing and aspiration are used independently, as in Hindi, than if they are not, as in French, and systems without independent voicing and aspiration are more common cross-linguistically (Dryer & Haspelmath 2013). Likewise, in the set of non-manual features of a sign language, if squint and brow raise are used independently, the system is formally more complex than a system in which squint and brow raise are not used independently (Dachkovsky et al. 2013). Sandler has argued that it takes time for the system to develop this independence among non-manual features in ABSL (Sandler, Meir et al. 2011). Equally important is determining where in the system increases in complexity first occur, and how this affects the inventory and the overall phonological space of a language.

Substantive bias refers to the degree to which phonological structures are physically grounded outside of the phonological system. There are two types of substantive bias. One is phonetic bias: phonological systems favour forms that are easier to produce and perceive phonetically. One way to see the effect of ease of production on spoken language phonological systems is by comparing vowel repetition and consonant repetition. Consonant repetition requires re-articulation of the articulatory gesture every time a consonant is re-produced (e.g. [pipopu]), while vowel repetition requires only that the gesture to produce the vowel be held in place over several syllables (e.g. [putuku]). Vowel harmony is therefore phonetically easier to produce than consonant harmony (Browman & Goldstein 1989, 1992), and is also more common cross-linguistically (Gordon 1998, Nevins 2010, Rose & Walker 2011); hence we see production bias at work. Perceptual bias is seen in systems that maximise the distance between elements in the phonetic/phonological space, as argued in Dispersion Theory (Flemming 2002, 2017). For example, if a system has just three vowels, the cross-linguistic tendency is for them to be as dispersed in the phonological space as possible: [i u a] are more dispersed in the vocal cavity than [i y e]. Likewise for handshape, low-complexity handshapes exhibit a phonetic bias. Handshapes with fully open 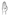 and fully closed 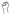 joint configurations, and those that use the whole hand 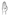 and the index finger 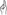 as selected fingers, are easier to produce (Ann 2006) and perceive (Lane et al. 1976) than medium- or high-complexity forms.

The second type of substantive bias is iconic bias. This type of substantive bias provides grounding of the phonological form with its referent in the external world. Although understudied in spoken languages, iconic forms in sign languages are well-studied and abundant. Iconicity plays a role in phonological and morphophonological patterns in sign language typology (Hwang et al. 2017), emergence (Abner et al. 2019), expansion of the lexicon (Occhino 2017), first-language acquisition (Caselli & Pyers 2017) and second-language acquisition (Ortega 2014). When iconic forms and phonetically easier forms are placed in competition with one another, the iconic forms often win out. For example, two-handed signs can drop (or delete) one of the hands, favouring ease of production (Battison 1974). The use of two hands to iconically represent meaning is well-attested (Lepic et al. 2016), and in cases where the two-handed form is iconic (hang clothes, meet, etc.), weak hand deletion is blocked in order to preserve iconicity (Brentari 1998). In spoken languages there are fewer ways that iconicity can apply to word-level meaning, but ideophones (Perniss et al. 2010, Dingemanse et al. 2016, Haiman 2018) and sound symbolism (Hinton et al. 1995, Shintel et al. 2006) demonstrate that iconicity may still be important in spoken languages, although the claim that iconicity is a bias in spoken languages has not yet been tested.

Iconic bias in handshape is important for this study, because object and handling handshapes display different types of iconicity. We might see different effects of bias in the iconicity associated with handling handshapes, i.e. when the hand represents how humans manipulate objects (hand-as-hand iconicity), or with object handshapes, i.e. when the hand represents the shape of objects (hand-as-object iconicity). Examples are given in (2).

-
  - Object handshapes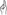 long thin object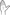 flat object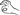 small round object
  - Handling handshapes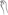 handle long thin object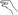 handle thick flat object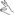 handle small object

The hand-as-hand iconicity seen in handling handshapes is used more readily in signs created on the spot by gesturers than the hand-as-object iconicity seen in object handshapes (Marentette & Nicoladis 2011). Previous work suggests that handshapes with high levels of selected finger complexity, seen in object handshapes, are used very little in co-speech or silent gesture (Brentari et al. 2012, Brentari et al. 2017; but see Janke & Marshall 2017).

### 1.3 Non-linguistic factors

Non-linguistic factors, such as community size and types of social contact, also affect the emergence of phonology. Typological and historical studies on spoken languages have suggested that a community’s size and social structure can affect the size and complexity of the phonemic inventory. Larger speech communities tend to have larger phoneme inventories, while smaller communities tend to have correspondingly smaller ones. This is referred to as the ‘founder effect’ (Atkinson 2011), because older linguistic communities tend to be larger. In independent studies, Atkinson (2011) and Hay & Bauer (2007) analysed 504 and 216 spoken languages respectively, using data from Dryer & Haspelmath (2013). They found that comparatively larger communities had larger vowel and consonant inventories, even when language family was considered as a factor. However, this finding has been challenged by a number of researchers, who suggest that the internal dynamics of linguistic communities also affect the size and complexity of a phonemic inventory (e.g. Lupyan & Dale 2010). For example, Bybee (2011), Maddieson et al. (2011) and Trudgill (2011) describe a tendency for more isolated, smaller communities to preserve complexity.^3^ Furthermore, iterative learning experiments would suggest that complexity decreases as the number of people providing input gets larger, and with more iterations of information transfer (Kirby et al. 2008, Smith et al. 2017, Kocab et al. 2019). Internal social factors could, therefore, be at least as important as the size of the community, or community size could be functioning as a proxy for several factors.

The analyses presented in this article may allow us to directly observe and tease apart the non-linguistic factors and their independent influence on the size and complexity of phoneme inventories. One of the sign language communities is relatively small and homogeneous (CTSL); the other is relatively large and diverse (NSL). Within each language community there are groups of signers (cohorts or linguistic generations) with limited or no access to a language model, while other cohorts have the benefit of a language model from an earlier cohort. By closely comparing these two emerging sign languages, we can isolate which factors are most important in particular circumstances.

To summarise, this study will analyse the handshape inventories of CTSL and NSL as they develop and create iconic, meaningful distinctions. No work to date has directly analysed comparable phonological data across two emerging sign languages as their phonology develops across cohorts. Our predictions are given in (3).

-
  - Handshape class (handling/object) will affect the size and complexity of handshape inventories.
    We expect high joint complexity in handling handshapes in all groups, while high selected finger complexity in object handshapes may be more restricted.
  - Both structural and substantive bias will be evident at the early stages of phonological emergence.
    Structurally simpler forms are expected to be most prevalent overall, and diachronically, we expect complexity to change in a non-linear fashion, due to the multiple factors involved.
  - Community size and type of contact among deaf members of the community will affect the size and complexity of the inventory.
    We predict a larger and more complex inventory in the larger community (NSL) than in the smaller community (CTSL).

## 2 Methods

This study is designed to analyse handshape features (joint configuration, selected fingers) and their use in two handshape classes (object, handling) in order to measure differences among cohorts and across languages, and to tease apart the effects of community size and contact. CTSL, a village sign language, has a relatively small number of deaf people within a community that includes signing deaf people and hearing people who do not use CTSL as their primary language and whose proficiency varies. The community has a relatively homogeneous sociocultural background and lives in an isolated, rural area. In contrast, NSL, a community sign language, has a relatively large number of deaf people from diverse backgrounds, and few hearing signers. Both sign languages are roughly the same age (approximately 50 years). In addition, we also study homesigners in Nicaragua, who provide insight into systems created by individuals who have little or no access to a sign language.

### 2.1 Participants

There were six participant groups: three from Turkey and three from Nicaragua. The CTSL participants were four first-cohort signers (aged 45–56), four second-cohort signers (aged 40–45) and four third-cohort signers (aged 16–22). The NSL participants were four first-cohort signers (aged 33–43) and five second-cohort signers (aged 21–26). We also included four Nicaraguan homesigners (aged 20–29). The total number of participants was 25; of these, 24 were deaf, and one was the hearing child of deaf CTSL parents who used CTSL as their primary home language. Naturally, signers from different cohorts of CTSL interact with one another, as do signers across NSL cohorts, as in any multigenerational community. We provide community profiles of the CTSL and NSL groups in the sections that follow.

#### 2.1.1 Central Taurus Sign Language.

CTSL is a village sign language that has emerged within the last 50+ years in an isolated mountainous area in the Central Taurus Mountain range of Southern Turkey. The residents of the villages are natives of central Anatolia, and the small gene pool has been preserved within the community for at least seven generations (Ergin 2017, Ergin & Brentari 2017, Ergin et al. 2018, Ergin et al. 2021). CTSL has developed in three small neighbouring villages, with little or no influence from Turkish Sign Language. The deaf individuals in these villages are connected to each other by birth and through marriage. Recessive deafness in the community and the prevalence of consanguineous marriages in families with deaf individuals have resulted in a high incidence of deafness (approximately 4.6% in the village from which the data discussed here were taken, compared to a typical incidence of deafness of approximately 0.5%). In addition to the deaf individuals, many hearing members of the community can sign CTSL, with varying proficiency. There are economic, geographical and cultural conditions that isolate the region. The community is located in one of the most mountainous regions of Turkey, and the villages are self-sustaining, with agriculture and animal husbandry the primary means of earning a livelihood.

The CTSL community is one large extended family, sharing history, beliefs, cultural practices and even a common family name. Marriage between first cousins is common, resulting in a multiplex of close kinship ties. Many of the people in the village know each other well, most know of each other and every deaf person knows every other deaf person in the village.

As deafness and sign language are inextricably woven into village society, CTSL is a viable language in the village, along with Turkish. Most hearing people in the village know deaf people, and are exposed to fluent communication in CTSL, used across a wide range of topics. Hearing children with deaf relatives acquire CTSL along with spoken Turkish. Deaf individuals take part in all social gatherings, work in fields with the other villagers and hold occupations similar to those of hearing members of the community. Deaf villagers are free to marry hearing or deaf community members.

Educational opportunities for hearing and deaf children in the village have been, and continue to be, limited. Education was neither compulsory nor readily accessible until the 1990s. There is currently an elementary school for hearing children in the village, but the nearest middle school is located in a town approximately ten miles away, and until the early 2000s was reachable only by foot or donkey, via a rugged mountain trail. Highways built since the early 2000s have made transportation easier for villagers and decreased the geographical isolation for hearing children; however, challenges for deaf children remain.

Because of social, geographic and financial obstacles, most hearing children do not attend school beyond the compulsory five-year education period, and deaf children typically do not attend school at all. Until the early 2000s, most deaf individuals in the village received no formal education. The closest elementary school for the deaf is 250 kilometres away, in Adana, and this is a formidable obstacle for many families.

We have learned through interviews that CTSL took hold during the fifth generation of the community’s existence. This fifth generation had twelve deaf members (later joined by four deaf spouses). Today, there are three living generations of deaf members in the village community (fifth, sixth and seventh), all of whom have contributed to the formation and maintenance of CTSL. CTSL1 signers are members of the fifth generation of the village community, and are the first deaf individuals born in their families; that is, they do not have older deaf people within their own family. They are termed Cohort 1 signers, because they had access to other deaf people outside of their immediate family, and therefore have had horizontal contact throughout their lives. Members of the two subsequent generations (the sixth and seventh), are referred to as CTSL2 and CTSL3 signers (Cohort 2 and Cohort 3 respectively). CTSL2 signers are the younger siblings of CTSL1 signers, and CTSL3 signers are the children of CTSL1 and CTSL2 signers. There is overlap in age across CTSL1 and CTSL2. CTSL 2 and CTSL 3 signers have engaged in both horizontal and vertical contact, and signers from these two cohorts have had contact with deaf members of their extended families since birth.

#### 2.1.2 Nicaraguan Sign Language.

NSL is approximately 45 years old. Before the 1970s, deaf Nicaraguans had little contact with each other (Kegl & Iwata 1989, Polich 2005, Senghas 2005, Senghas et al. 2005). There were periods when various classrooms and clinics were available to young children, but the lack of a unifying national educational system, societal attitudes that isolated deaf individuals, and marital patterns that generally precluded hereditary deafness prevented intergenerational contact and formation of a deaf community. However, one school, founded in Managua in 1974 with 25 deaf students, expanded to include 100 students in 1979 when it became more publicly accessible. The following year, a vocational school opened for adolescents. By 1983, the schools served more than 400 deaf students (Polich 2005). For the first time, a community existed with continuity from childhood through early adulthood. There is now a Nicaraguan Deaf Association, and NSL signers in Managua see each other frequently.

Community sign languages define, at one and the same time, both deaf communities and signing communities. Members of these communities meet in schools, in deaf clubs and in social gatherings, but they do not necessarily share a restricted geographical area or single social background. The signers who catalysed the emergence and development of NSL are characterised by a higher degree of heterogeneity than the signers of CTSL (Coppola 2020).

The NSL community of today has been divided into cohorts based on their year of entry into the signing community. The first cohort of signers began life as homesigners, were brought together as children and formed a deaf community in the late 1970s and early 1980s.^4^ These individuals produced and saw the signing of others in their environment at that time, and the resulting variety of the language is called the ‘initial contact variety’, or first-cohort signing. We refer to this group as NSL1 (Senghas et al. 2005). NSL1 signers therefore have horizontal contact, since they signed with each other, but they had no model from whom the language could be learned. A second cohort of signers (referred to here as NSL2 signers) are deaf individuals who entered the school, and *de facto* the community, in its second decade, from the mid-1980s to the early 1990s. They had access to teenagers already at the school who were NSL1 signers. NSL2 and subsequent cohorts (NSL3, NSL4, etc.) are said to use a ‘sustained contact variety’. These signers have vertical contact in addition to horizontal contact, since in addition to a shared, deaf signing community, they had access to the signing produced by the previous cohorts as a language model.

The majority of deaf individuals in Nicaragua are not part of this signing community, and do not know NSL; due to a variety of social, geographic and financial obstacles, they do not go to school or interact with other deaf people. These deaf individuals are known as ‘homesigners’, and represent the sign systems that fed into the creation of NSL. The homesigners included in this study have hearing losses that are significant enough to prevent the acquisition of a spoken language, and they have not had regular exposure to NSL or much formal education. None has successfully learned written or spoken Spanish. These homesigners do not know each other, do not have regular interactions with deaf or hearing signers of NSL and have been using their individual homesign systems as their primary language for their entire lives (Coppola 2002). Each homesigner has a unique communication history with hearing family members and friends with whom they interact regularly (Coppola 2002). While their hearing communication partners engage the homesigners using gestures, the homesign system is not shared among them at the lexical (Richie et al. 2014) or syntactic (Carrigan & Coppola 2017) level. This lack of a shared system is important, because it means that the homesigners have neither horizontal contact (interactions with peers using a shared system) nor vertical contact (with previous cohorts). Essentially, they form a linguistic community of one person, producing, but not receiving, an individual homesign system.

#### 2.1.3 Classification of participants.

The CTSL community is divided into cohorts based on kinship: CTSL1 signers are the first deaf members of their families, CTSL2 signers are the younger siblings of CTSL1 signers and CTSL3 signers are children of CTSL1 and CTSL2 signers. CTSL2 and CTSL3 signers have sign language within their families from birth, although evidence for ‘familylects’ is weak (Ergin et al. 2021).

The NSL community is divided into cohorts based on their year of entry into the school for special education, as they were first exposed to sign language when they entered school. NSL1 signers were brought together at the school in Managua as children in the late 1970s and early 1980s. NSL2 signers entered the school from the mid 1980s to the early 1990s. They had access to teenagers already at the school, who were NSL1 signers.

Despite the different circumstances, the type of contact is comparable across the two languages, and the participant groups can be classified with respect to their access to a community of users, as in Table I.

Homesigners have a community of one person, and little or no access to other signers. The members of all five remaining groups have horizontal (H) contact, i.e. exposure to other signers. Two groups, CTSL1 and NSL1, have only horizontal contact (+H, −V). Three of the groups, NSL2, CTSL2 and CTSL3, also have vertical contact; i.e. exposure to an existing language model from the previous generation of signers, along with the contact with their own cohort (they are +H, +V).

We also analyse the effect of community size on the development of handshape complexity. Here ‘community’ refers to those individuals who use the system as their primary means of communication; thus we report only the number of deaf signers in each group, not hearing signers. As noted above, homesigners have a linguistic community of one. CTSL signers come from a relatively small community of 5–10 deaf signers in CTSL1, and 15–25 deaf signers in CTSL2 and CTSL3. NSL signers come from a relatively large community, with between 300 and 400 deaf signers in NSL1, and 300 deaf signers in NSL2. Additional deaf signers in subsequent cohorts have since joined the community, comprising a community of approximately 1500 altogether (Senghas 2019).

### 2.2 Material and procedures

#### 2.2.1 Stimuli.

The stimulus items were drawn from a set of 121 photographs and short videos (henceforth vignettes), each of which featured one of eleven objects designed to elicit handling and object handshapes in a variety of event structures (singular, plural; agent, no-agent). The objects in the stimulus clips exhibit a typical range of colours, shapes and sizes. We confine our analyses to 88 trials featuring eight object types collected from every participant: toy planes, books, coins, lollipops, marbles, pens, television sets and tweezers. Each object was portrayed in eleven conditions: five depicted a stationary object or an object moving on its own without an agent, five depicted an object being moved by the hand of an agent and one depicted the object as it is typically used.

The stimulus objects and vignettes that are included in the task represent a balance of iconicities regarding size and shape (to elicit object handshapes) and how objects are handled (to elicit handling handshapes). Because handshape inventories are typically skewed toward simple handshapes (Hara 2003, Eccarius 2008), we included several objects chosen to facilitate the use of handshapes with medium- and high-complexity selected finger or joint configuration (e.g. toy planes, marbles, television sets and tweezers).

#### 2.2.2 Procedures.

Signers were instructed in their respective sign language or homesign system to watch each video and describe what they saw to an interlocutor. The instructions provided to all groups were minimal, because the elicitation task was quite straightforward and did not require elaborate instructions. This procedure also accommodated the homesigners. For CTSL signers, the interlocutor was a family member. For the Nicaraguan groups, the interlocutor was a familiar communication partner for the homesigners, or a peer from the same cohort for the NSL signers. Data collection sessions were videotaped, and the video files containing the participants’ responses were transcribed using ELAN (Crasborn & Sloetjes 2008).

### 2.3 Transcription

Annotation was performed under the supervision of the first author. The NSL data were annotated at the University of Chicago, the CTSL data at Tufts University and the homesign data at the University of Connecticut. All annotators had at least 50 hours of training on the coding system, until they reached a threshold of reliability of 90% with a standard set of training items on the properties under investigation, described below.

#### Label vs. event.

We segmented responses into signs used to label the object (typically those produced on the body or in neutral space, i.e. without reference to a specific location) and signs used to describe the event or spatial arrangement shown in the vignette (those that moved or were situated at a particular location).^5^ Video examples are provided here for toy plane, produced by a CTSL3 signer, and marble, produced by a NSL2 signer. This distinction could be made without difficulty, given that we were using a controlled task. There was 96% intercoder reliability for label vs. event descriptions.

#### Handshape class and specific handshape.

Each handshape for both labels and event descriptions was annotated for its handshape class: handling handshapes represented the manipulation or handling of the object, object handshapes represented the size or shape of the whole object or part of the object and other handshapes were neutral handshapes used to trace the object’s path or indicate its location. The specific handshape was also annotated. Annotators chose the best match for the produced handshape from among 100 possible handshapes collected and annotated for joint and selected finger features during several previous cross-linguistic studies conducted by the first author (Eccarius & Brentari 2007, 2008, Brentari et al. 2012, Brentari et al. 2015, Brentari et al. 2017). Reliability was assessed on data for one stimulus object (e.g. all plane items) from one participant from each group. Agreement between annotators was at least 97% for handshape class and 91% for specific handshape form.

The feature annotations for each handshape were then assigned separate joint and selected finger complexity scores of low (1), medium (2) or high (3), as described in §1.1. In addition to the complexity level, a sign was given an extra point for complexity if there was a change in the joint, selected finger features or both during the sign’s production. For example, if the handshape changed from a flat-open to a flat-closed handshape, an extra point was added for joint complexity, or if the handshape changed from an index finger to a handshape using all of the fingers, an extra point was added for selected finger complexity. The total complexity for each participant is the average of joint configuration and selected finger complexity. Thus complexity values for joint configuration and selected fingers ranged from 1 to 4.

## 3 Analysis

The handshapes and associated features produced by participants to express the labels and events in their vignette descriptions were analysed qualitatively and quantitatively. A total of 1992 vignette descriptions were produced by the 25 participants in response to the 88 items; all signers responded to all items. Signs produced with handshapes that could not be categorised as handling or object – i.e. ‘other’ handshapes and productions with both handling and object handshapes – were excluded from further analysis (6% of the total dataset). 5318 handshapes were used in both the qualitative and quantitative analyses.

### 3.1 Innovative method for determining phonemic status

**Figure 4:**
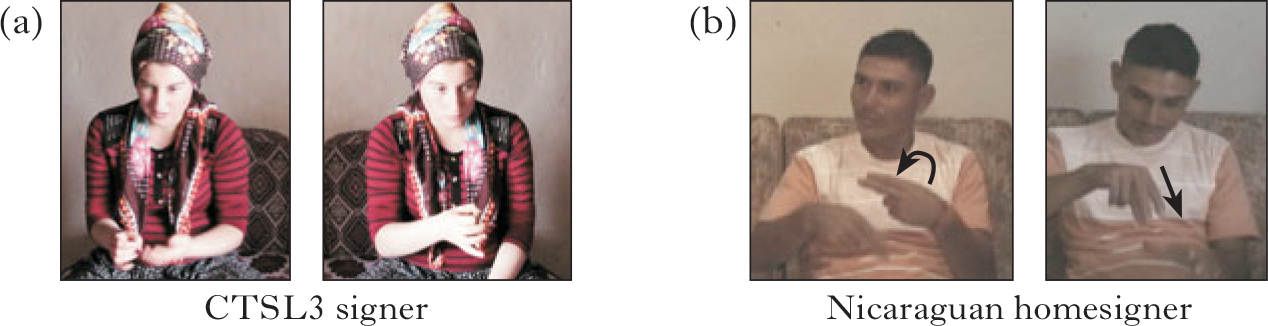
(a) A CTSL3 signer using the same curved-closed joint configuration in two different selected finger groups: the B-handshape (left) and the 1-handshape (right); (b) a homesigner using the same selected finger group (the U-handshape) in two different joint configurations, extended (left) and spread (right).

As noted in the introduction, while minimal pairs constitute a typical test for inclusion of a phonological element in a phonemic inventory, CTSL and NSL apparently have no minimal pairs, because other features in the sign (i.e. location and movement) also vary when joint or selected finger features vary. To allow the investigation of the state of a system without evident minimal pairs, the notion of the minimal pair was relaxed as follows. A feature for joints or selected fingers was included for a participant if it was used in two different handshapes in the other feature class (for example, a selected finger feature used in two different joint configuration classes, or a joint configuration feature used in two different selected finger classes). If a feature met this criterion for at least two individual members of a group, we included the feature in that group’s system. An example produced by a CTSL3 signer in Fig. 4a shows the same joint configuration feature (curved-closed) used in two different selected finger groups, the B-handshape and the 1-handshape. The non-manual behaviour is different as well. In Fig. 4b, produced by a homesigner, the same two-finger selected finger group is used in two different joint configurations, extended and spread. The movement is also different (curved vs. straight).^6^

### 3.2 The effect of complexity on handshape class

**Figure 5:**
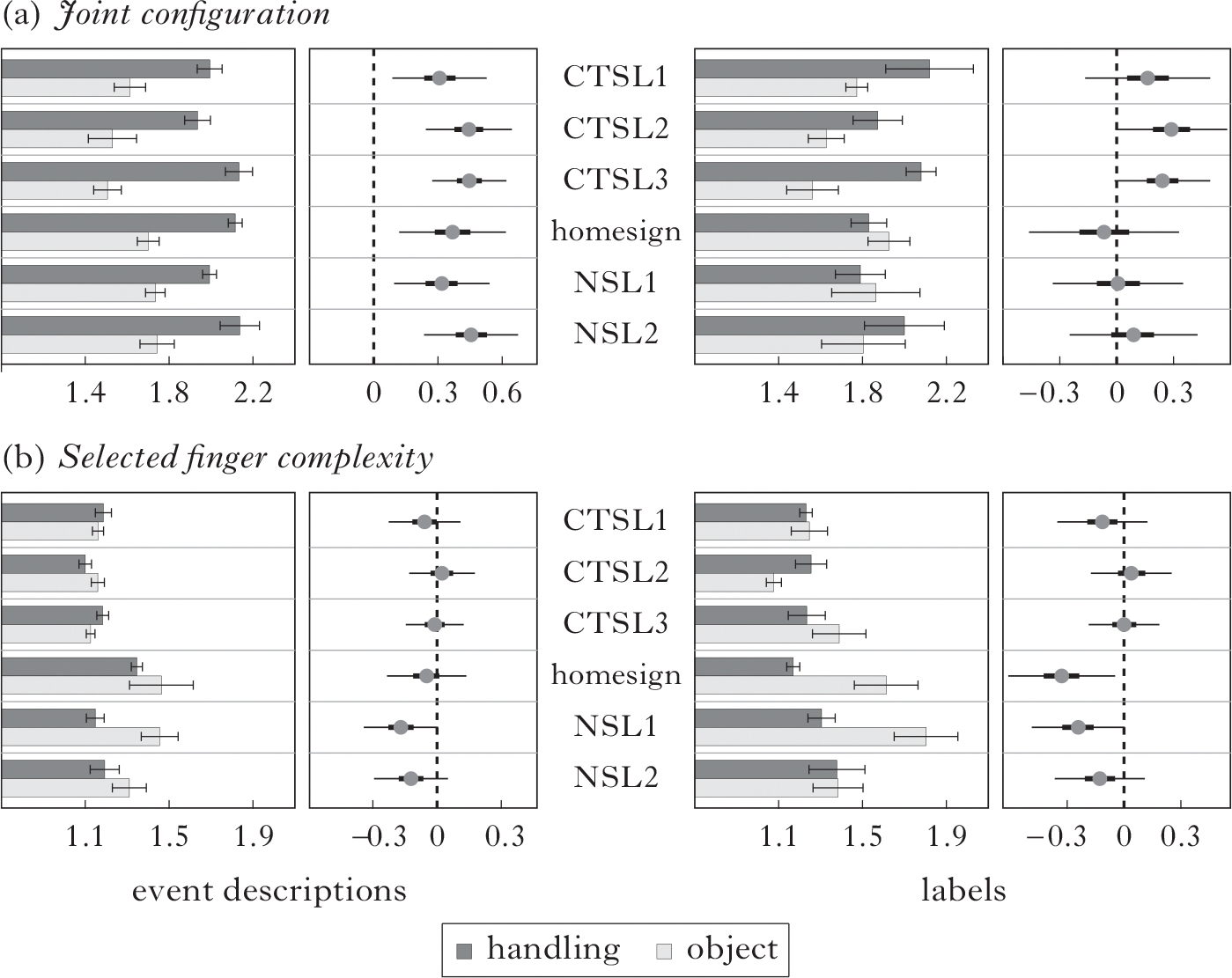
Results for differences in (a) joint configuration and (b) selected finger complexity across handshape class (handling, object) in event descriptions (left) and labels (right). Horizontal bar graphs show average level of joint configuration and selected finger complexity by group and handshape class (with standard error bars). On the right of each bar graph is the probable difference between handshape classes (handling minus object handshape complexity), showing means (grey circles), 50% credible intervals (thick lines) and 95% credible intervals (thin lines).

The first prediction is that handshape class (handling or object) will affect complexity, and this prediction is confirmed. In Fig. 5 the data is presented by group and handshape class, with event descriptions and labels presented separately. The bar graph on the left of each panel shows the average complexity levels for joint configuration (a) and selected finger features (b) in event descriptions, by group, for handling and object handshapes. To the immediate right of each bar graph, we present the output of a Bayesian multilevel model (*brms* package), which tests the reliability of the observed differences (Bürkner 2017). On the right of the figure, the corresponding data and Bayesian model output is shown for labels.

A Bayesian model was used because neither joint configuration nor selected finger measures have a normal (bell-curve) distribution, and Bayesian analyses do not assume a normal distribution, unlike other more commonly used tests, e.g. *χ*^2^ or ANOVA. Instead of a *p*-value for each measure, a Bayesian analysis provides a credible interval, which expresses a belief about the effect size. Here we apply the following common heuristic: we set the credible intervals to 50% and 95%; 95% is roughly equivalent to *p* ≤ 0.05. If a factor exceeds a credible interval of 95%, the thin horizontal line does not cross the vertical line labelled ‘0’, and we can be reasonably sure that the measure is predictive of the result. The model included random intercepts for Participant, Item and Vignette, and random slopes for handshape class and for within-item factors.^7^ (See the Appendix for individual data.^8^)

As can be seen in Fig. 5, handshape class affects complexity, as predicted, and so does group, which we explore further in the next section. In every group’s event descriptions, handling handshapes have higher average joint complexity than object handshapes. In CTSL2 and CTSL3, we see this difference in labels as well (in Fig. 5a, right, the 95% interval touches but does not cross ‘0’). In NSL1, for both event descriptions and labels, object handshapes have a higher average selected finger complexity than handling handshapes (Fig. 5b, left, right). The homesigner group also shows this difference in selected finger complexity, but only in labels (Fig. 5b, right). Thus there is a strong iconic bias for handling handshapes to have relatively high joint complexity, particularly in event descriptions, and a weaker iconic bias for object handshapes to have relatively high selected finger complexity, particularly in labels (NSL1 and homesigners).

### 3.3 Phonemic inventories by group

We now present the inventories of each group, taking as our starting point the handshapes provided in Fig. 2, which represents the range of joint configuration and selected finger handshape groups attested in the current dataset. First we present the inventory used by all groups, then by the CTSL groups, and lastly by the Nicaraguan groups.

**Figure 6:**
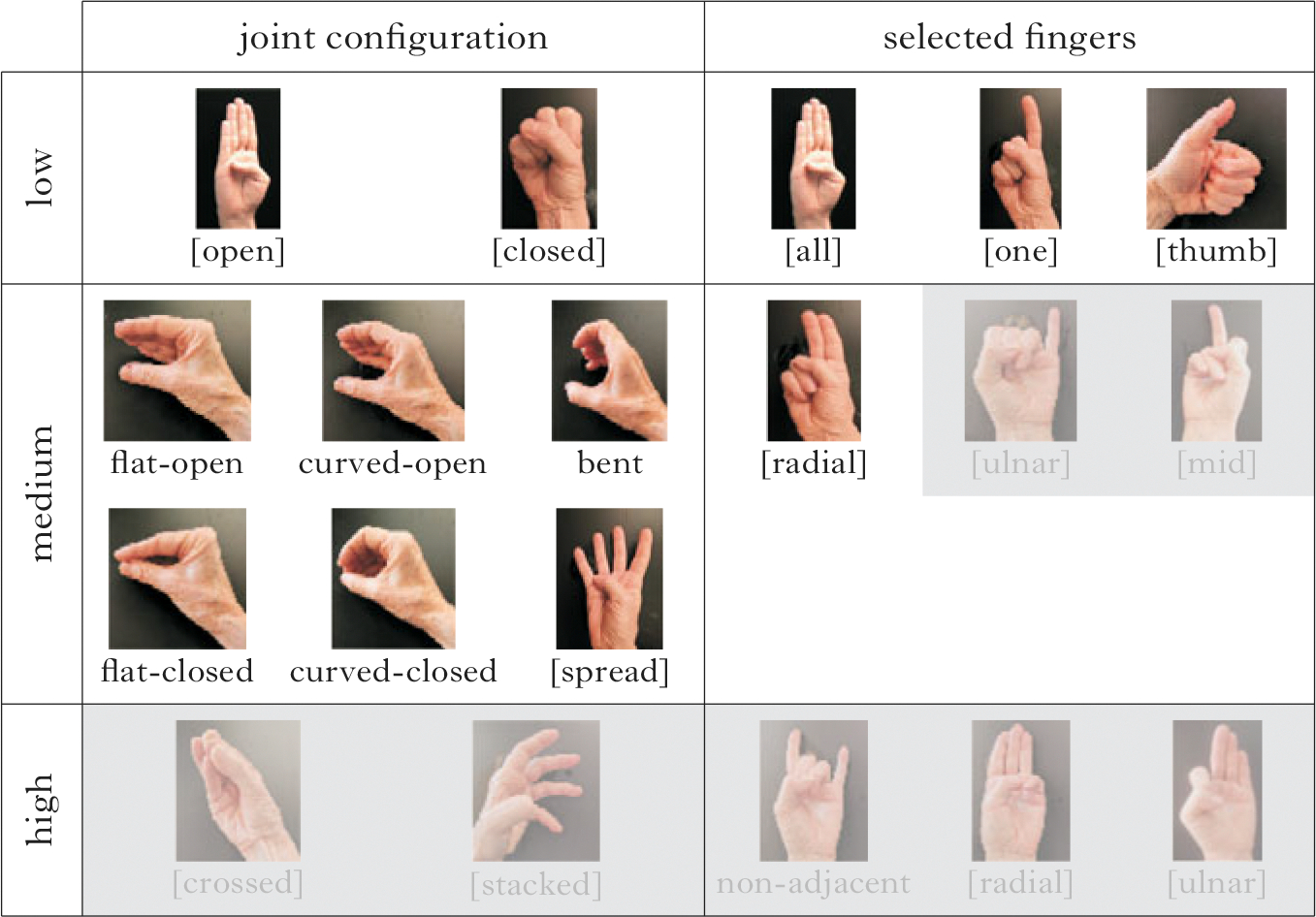
Feature inventory for all groups in the entire dataset; features not used are shaded.

Figure 6 shows the subset of features used in the inventories of all the groups. Combinations of these features resulted in an inventory of 27 handshapes. Features not used are shaded. There are no high-complexity feature combinations in the set of handshapes used by all groups.

**Figure 7:**
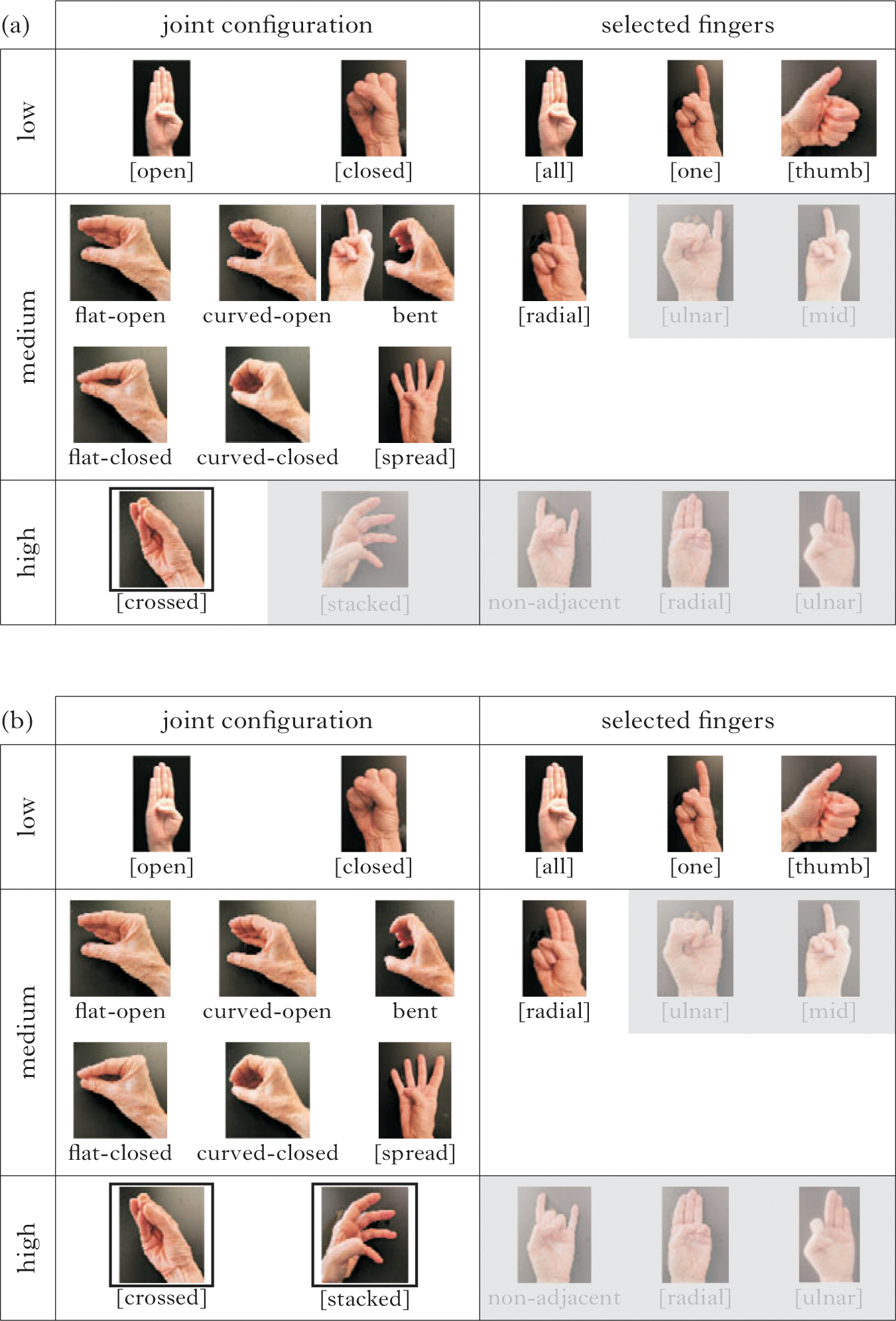
Feature inventory for (a) CTSL1, and (b) CTSL2 and CTSL3. Features not used are shaded; black boxes indicate added features compared to the set used by all groups.

Next, we examine the inventories across the CTSL groups (Fig. 7). The features used in the CTSL cohorts are similar to the common set of features in Fig. 6, and the inventories are very similar to each other. Combinations of joint configuration and selected finger features in CTSL1 result in an inventory of 36 handshapes. In CTSL1 (Fig. 7a) one handshape group is added, which uses the high-complexity joint configuration feature [crossed].

Figure 7b shows the features used by CTSL2 and CTSL3 signers; the two groups have identical feature sets. One additional high-complexity handshape group beyond the CTSL1 inventory is added, which uses the high-complexity joint feature [stacked]. Combinations of joint configuration and selected finger features result in an inventory of 37 handshapes. Note that the feature additions in CTSL, beyond those of Fig. 6, are along the dimension of joint (not selected finger) complexity.

**Figure 8:**
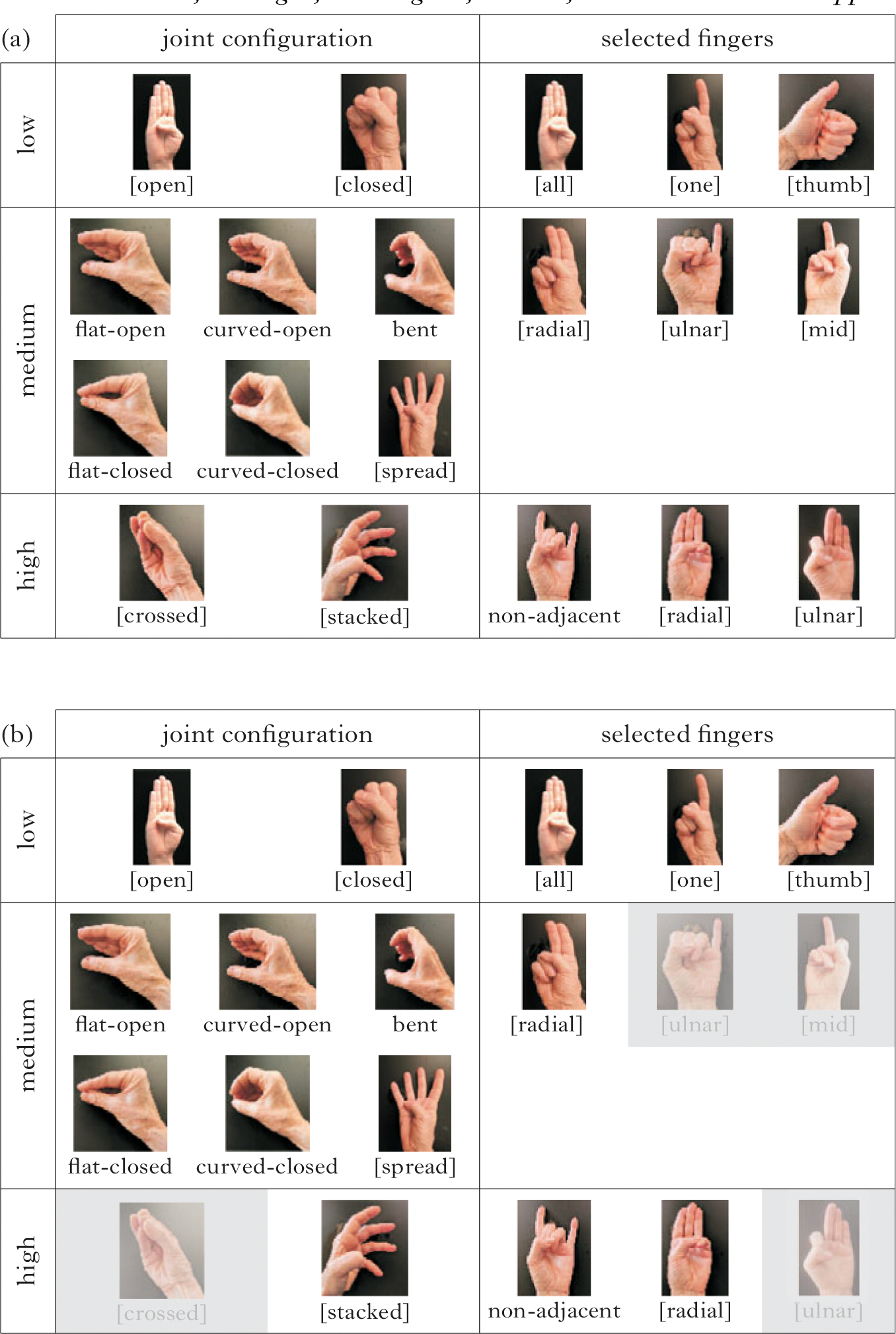

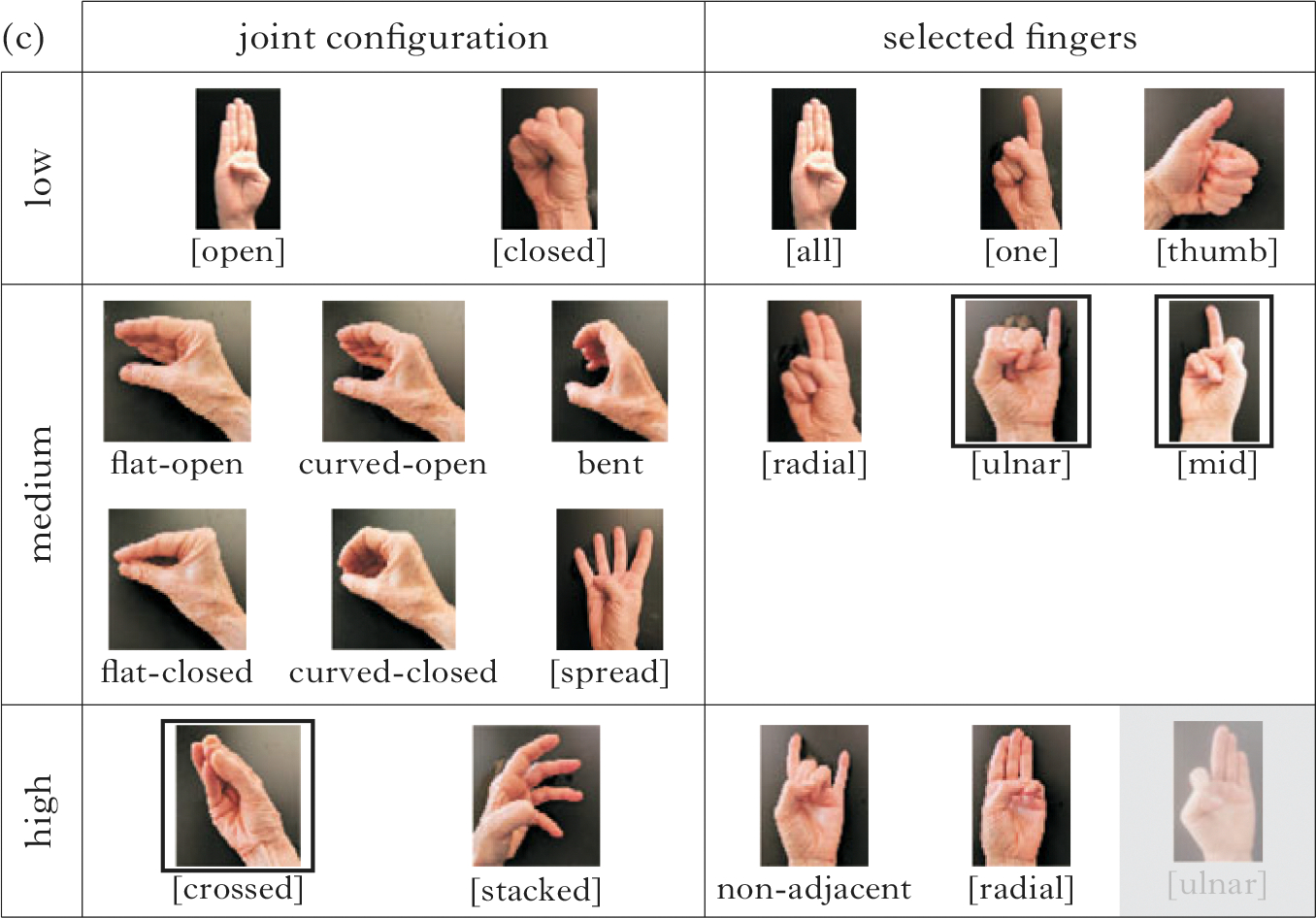
Feature inventory for (a) homesign, (b) NSL1 and (c) NSL2. Features not used are shaded; black boxes in (c) indicate features that have been reintroduced in NSL2.

We now turn to the feature inventories of the Nicaraguan groups. The homesign group shows the largest and most complex inventory of the six study groups. The homesigners use all of the handshapes represented in Fig. 2, and feature combinations of joints and selected fingers result in an inventory of 54 handshapes (Fig. 8a). It is important to note that each homesigner had a complex handshape inventory (see the Appendix for data by individual signer).

In the NSL1 inventory (Fig. 8b), the number of handshape groups is reduced by four feature groups with respect to the homesign inventory. The joint configuration feature [crossed] is missing entirely, and the point of reference features [mid] and [ulnar] are missing in combination with one- and three-finger groups. Combinations of joint configuration and selected finger features result in an inventory of 31 handshapes for NSL1.

One plausible motivation for the reduction from the homesign inventory to the NSL1 inventory is that when signers are both producers and perceivers of the system, as the NSL1 signers are, the inventory is scaled back. The gaps in NSL1’s inventory seem to be an attempt to thin out the clutter in the middle of the complexity scale, while maintaining the full range of the phonological space (there are still low-, medium- and high-complexity handshapes). Recall that all of the NSL1 signers went to the school in Managua as homesigners, so this change has happened in less than one lifetime.

The NSL2 inventory (Fig. 8c) reinstates three of the four feature groups that the NSL1 took away: the [crossed], [mid] and [ulnar] groups are now back in the inventory; however, the group with three selected fingers with an [ulnar] point of reference is not reinstated. Combinations of joint configuration and selected finger features result in an inventory of 36 handshapes for NSL2.

The inventories reveal several things. First, we observe that the inventories of CTSL are more stable than those of the Nicaraguan groups. CTSL1 differs from CTSL2 and CTSL3 in only one handshape group, and the CTSL2 and CTSL3 features do not differ at all. In contrast, the changes across the groups in Nicaragua are more dramatic. Given the way that the CTSL signers are divided into cohorts, this uniformity might be due to an overlap in age, as we see in CTSL1 and CTSL2. However, the clearer age difference is between CTSL2 and CTSL3, who have identical inventories, not between CTSL1 and CTSL2, where the ages overlap.

Second, we see support for structural bias at work, since the group of 27 handshapes used by all groups employs (at most) medium levels of complexity; there are no high-complexity forms. We also predicted that across cohorts, changes in complexity would not be linear, and we see that this is the case. Homesigners, both individually and as a group, produce larger inventories than those found in either CTSL or NSL. This suggests that having a ‘community’, with horizontal (NSL1) or vertical (NSL2) contact, reins in the inventory to a more manageable size, perhaps to increase the perceptual distance between forms.

Third, the prediction that the larger community will have a larger and more complex inventory is only partially supported. The inventories in CTSL and NSL are relatively equal in size; however, the Nicaraguan groups use a greater range of selected finger features than the CTSL groups do. There is greater complexity in NSL, but not larger size. We investigate this question further in the next section.

### 3.4 The effect of non-linguistic factors on handshape inventories

**Figure 9:**
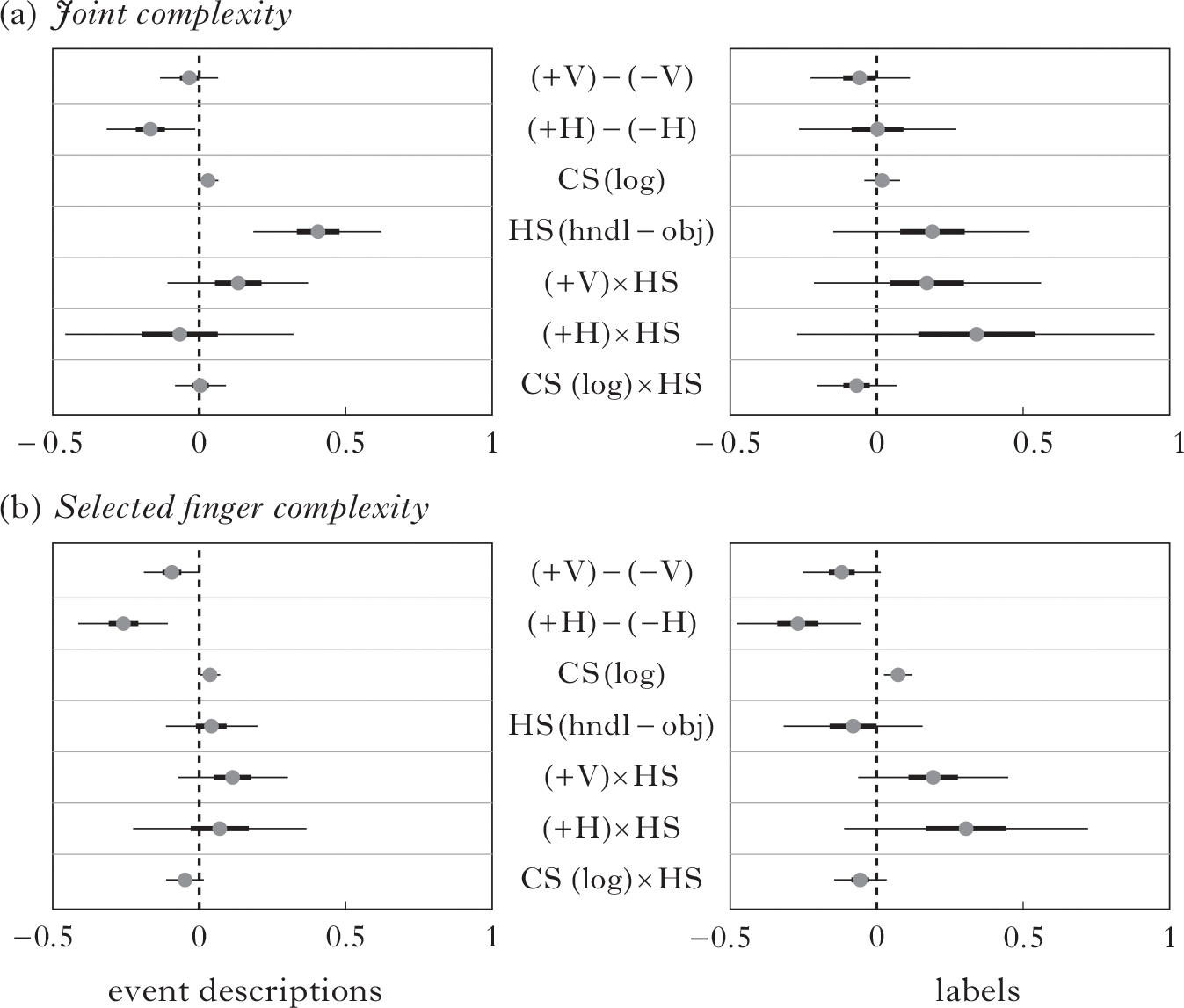
The posterior probability distributions of model parameters with means (grey circles), 50% credible intervals (thick lines) and 95% credible intervals (thin lines), for event descriptions (left) and labels (right) on measures of (a) joint complexity and (b) selected finger complexity. The *x*-axis gives the effect size.

In this section we analyse how community size and type of contact affect the complexity of the handshape inventories. We again employed a Bayesian model for the statistical analysis, because multiple factors interact, and because the data of the current study do not have a normal (bell-curve) distribution. We fitted factors of joint configuration complexity and selected finger complexity to a linear mixed-effects model. The analysis combines the data from all of the groups.^9^ The factors analysed are listed along the y-axis in Fig. 9, which displays the output of the model for each factor: horizontal contact (+H, −H), vertical contact (+V, −V), handshape class (handling, object) and logarithm of community size (i.e. number of deaf people in the community), along with the interactions of the three main predictors as fixed-effects terms.

We log-transformed community size to make the scales of the CTSL groups (25–30) and the NSL groups (approximately 1500) comparable, so that the statistical models would converge. In addition, as before, the model included random intercepts for Participant, Item and Vignette; and random slopes for Handshape class and within-item factors were also included. All of the categorical variables were effect-coded such that Vertical contact, Horizontal contact and Handshape class were set to a possible range of between 0.5 and −0.5. The homesigners were used as the reference group, since they have a community size of one, and have neither horizontal nor vertical contact; they thus serve as a reasonable baseline. If a factor exceeds a credible interval of 95% and does not cross the vertical line labelled ‘0’, we can be reasonably sure that the factor is predictive of the result.

Event descriptions (Fig. 9, left) and labels (Fig. 9, right) were analysed separately. In the analysis of joint configuration (Fig. 9a), three factors are predictive in event descriptions. Horizontal contact (HC; +H lower complexity than −H) is predictive, indicating that having a community constrains the number of feature combinations used (i.e. the change from Fig. 8a to Fig. 8b; the homesign group vs. NSL1). Community size (CS) is also predictive (a larger community has higher complexity), as also shown by the inventories described in Figs 7 and 8. In addition, Handshape class (HS) is predictive (handling handshapes have higher complexity). There are no predictive factors for labels.

In the analysis of selected finger complexity, two factors are predictive in both event descriptions and labels (Fig. 9b): Horizontal contact (+H lower complexity than −H) and Community size (a larger community has higher complexity).

The results in Fig. 9 suggest that a larger community size results in a more complex system, while, across cohorts in Nicaragua, contact of either type (horizontal or vertical) results in a less complex system. Note that the significant decrease in complexity comes between homesign and NSL1; the difference between +V and −V is not predictive. These results support the third prediction, that community size and contact among signers affect the *complexity* of the handshape inventory; however, recall from §3.3 that the inventory *size* is similar in CTSL and NSL.

To summarise the findings described in §3.2–§3.4, we have observed that the groups deployed features in the two handshape classes differently, supporting the prediction that Handshape class affects the inventory. Joint complexity and selected finger complexity do not have the same patterns across labels and events, or across groups. Change is not linear. The association of high joint complexity with handling handshapes in event descriptions is robust in all groups, but the association of high selected finger complexity with object handshapes is present primarily in the homesign and NSL1 groups. The analyses support the second prediction, that structural bias influences the common handshapes observed across groups, since the set of common handshapes (Fig. 6) has no high-complexity handshapes. Regarding the third prediction, the larger community, NSL, displays more complexity than the smaller community, CTSL. However, the amount of complexity decreases with contact among members – both horizontal and vertical – suggesting that any type of community reins in the size and the complexity of the inventory.

## 4 Discussion

Our results indicate that handshape class, learning bias, and community size and contact shape the development of a phonological inventory in an emerging language. CTSL and NSL show different patterns of phonological emergence in several ways.

### 4.1 Use of the phonological space

One important result of this study is that the NSL groups make more extensive use and more balanced use of the whole phonological space, exploiting both joint configuration and selected finger features, while CTSL employs primarily joint features. We illustrate this using the dimensions of the phonological space for obstruent consonants in spoken languages.

**Figure 10:**
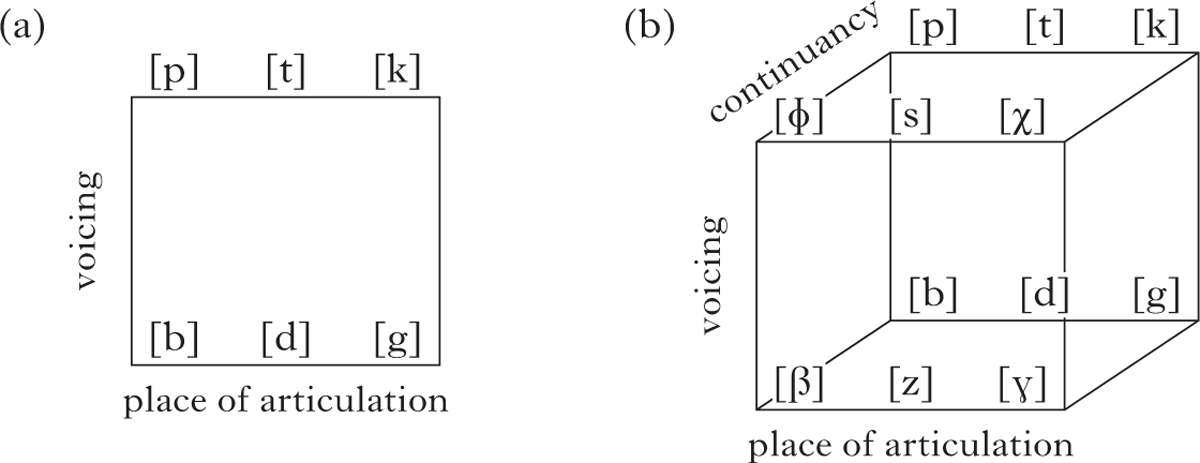
Hypothetical consonant inventories: (a) on two dimensions (place of articulation and voicing); (b) on three dimensions (place of articulation, voicing and continuancy).

The general trend is that in order to satisfy constraints which place a minimum on perceptual distance between segments (MinDistance) as well as increase the number of segments available (MaxContrast), as proposed in Dispersion Theory (Flemming 2002, 2017), languages can engage new dimensions within the phonological space. In Fig. 10a we see a hypothetical consonant inventory utilising two dimensions – place of articulation and voicing – while Fig. 10b is a consonant inventory with three dimensions – place of articulation, voicing and continuancy (cf. Dunbar & Dupoux 2016). With the addition of the feature [±continuant] to create the stop/fricative contrast, the inventory in Fig. 10b has the potential to double in size by adding a [+continuant] form to each of the [−continuant] forms.

**Figure 11:**
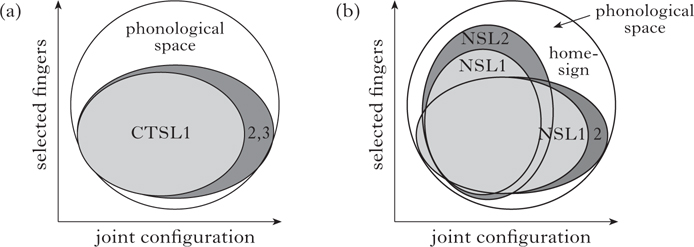
(a) The handshape space and distribution in CTSL. (b) The handshape space and distribution in NSL and homesign.

We can similarly visualise the phonological space for sign languages, where either the joint dimension alone or the two dimensions, joints and selected fingers, can be employed to fill the phonological space. The CTSL groups primarily use joint configuration features (Fig. 11a), while the Nicaraguan groups (Fig. 11b) exploit the space more fully by using both the joint configuration and selected finger dimensions to a greater extent, increasing the potential to increase the perceptual distance between forms and to add forms in the future.

### 4.2 Form–meaning mapping: iconic bias

It was noted in §1 that both CTSL and NSL communities use object handshapes more frequently in no-agent descriptions, and handling handshapes more frequently in agentive descriptions (Goldin-Meadow et al. 2015, Ergin & Brentari 2017), so signers from both languages show a morphosyntactic functional distinction between these two handshape classes. This morphosyntactic distinction does not, however, speak to how it is expressed in phonology. We asked whether emerging handshape inventories would move towards the double dissociation pattern seen in Fig. 3, in which handling handshapes are associated with high joint complexity and low selected finger complexity, and object handshapes are associated with high selected finger complexity and low joint complexity. Only the homesign and NSL1 groups show any evidence of this. Homesigners and NSL1 signers use relatively high joint complexity in handling handshapes in their event descriptions, as do all the groups. Both groups also show relatively high selected finger complexity in object handshapes – homesigners do so in labels, and the NSL1 signers do so in both event descriptions and labels.

In order to track the development of a phonological system, it is important to know where and when the complexity occurs. We see that all groups use all of the medium-complexity features for joint configuration, particularly in event descriptions, which function as verbal forms. This can be interpreted as an action bias, i.e. the accessing of a type of iconicity via action and action on objects (Piaget 1962), and is in accord with work on gesture in children (Marentette & Nicoladis 2011) and adults (Brentari et al. 2012, Brentari et al. 2017). The results of the current study strengthen the claim that hand-as-hand iconicity is a direct, relatively transparent and ubiquitously robust way to inject important iconic properties into a sign language phonological system in verbs. Object handshapes and their associated high selected finger complexity might also be interpreted as reflecting an iconic bias – an object bias – particularly in homesign, as seen in Fig. 5, where relatively high-complexity selected finger features are associated with object handshapes, particularly in labels, which function as nominal elements in the participants’ signed descriptions.

A phonological feature (or set of features) does not have to be used contrastively across a whole language; however, we might consider broadening the use of a feature from a more restricted, iconic context to a more general, less iconic one to be one indication of an emerging phonological system. Thus, extending the use of features in event descriptions *or* labels to events *and* labels shows a language-wide pattern of generalisation. Abner et al. (2019) make this same argument for nominalisation: the use of repetition to distinguish nouns and verbs becomes systematic when it is broadened from iterative contexts (iconic for repetition) to non-iterative events (not iconic for repetition). Thus, evidence for the emergence of phonology comes from CTSL2 and CTSL3 signers generalising the handshape class distinction in joint complexity to both labels and event descriptions, and from NSL1 signers generalising the handshape class distinction in selected finger complexity to labels and descriptions (see Fig. 5).

### 4.3 The effect of structural and phonetic bias

The results show robust evidence of structural and substantive bias (Moreton & Pater 2012a, b), but the story is complicated. The set of handshapes used by all groups is heavily weighted towards handshapes with simpler structures (i.e. structural bias) and towards those that are easier to produce and perceive (Lane et al. 1976, Ann 2006; i.e. substantive bias). But this is not so at the very beginning. Homesigners have a large and highly complex inventory, so structural and phonetic biases do not appear until there is a community of signers.

We also acknowledge that the structures illustrated in Fig. 1 already have considerations of phonetic bias built into them, so using an abstract feature geometry does not really isolate structural bias from substantive bias.^10^

### 4.4 The effects of community size and type of contact

This study has hypothesised that the size of the community and the patterns of contact among its members affect the size and complexity of the phonemic inventory. Leaving aside the homesigners for the moment, the inventories of the NSL groups (the larger, more diverse community) are not larger than those of the CTSL groups (the smaller, more homogeneous, more isolated community); however, the handshapes in the NSL inventories have higher complexity than those in CTSL (Fig. 8c; see also the Appendix). Importantly, we did not find evidence that the larger linguistic community (NSL) had a larger inventory, as reported in the spoken language typology literature (the ‘founder effect’; Atkinson 2011). The current study concurs instead with research suggesting that the factor of community size is a composite of many factors, and that other properties associated with community size may influence inventory complexity (Lupyan & Dale 2010, Maddieson et al. 2011). As noted, the CTSL community has more shared cultural and social knowledge than the NSL community. The CTSL community also has many more hearing signers who use the system, but not as their primary means of communication, which may also hamper the use of complex forms.

The results of the present study confirm the hypothesis that contact among signers affects the complexity of the handshape inventory; complexity decreases with contact, particularly in NSL. It is also important to keep in mind, however, that the effect of contact goes in the opposite direction from that of community size. With larger community size, complexity tends to increase, but with contact among signers, it tends to decrease. All CTSL and NSL groups have less complexity than homesigners. We suggest that the decrease in complexity is motivated, at least in part, by ease of perception, since the groups with contact have genuine two-way communication within a community, and thus produce and perceive the system.

One body of work that may be relevant here comes from iterative learning experiments (Kirby et al. 2008, Smith et al. 2017, Kocab et al. 2019), which demonstrate that input from a single person results in a more complex and varied set of output forms than input from multiple people. This aligns with the result that the most isolated individuals, homesigners (−H, −V contact), have the largest inventory and the most complex handshapes of all groups (Fig. 8a). We are, however, cautious about drawing parallels between iterative learning studies and those of the current study, since the data reported here occurred in a more natural setting, and have more degrees of freedom than those obtained in a laboratory. Also, all of the participants in iterative learning experiments have a preexisting language, while this is not the case in the emerging languages discussed here.

We might ask, had we been able to study homesigners in the villages where CTSL is used, whether they would have used handshapes with high selected finger complexity, perhaps as high as the Nicaraguan homesigners. Work by Horton (2018) found that peer homesigners (those with contact with other homesigners at school who have diverse backgrounds) had more selected finger complexity than family homesigners (homesigners who have another homesigner in their extended family with more shared experience). This suggests that other aspects of the social context may influence homesigners’ inventories; we leave this question for future research.

## 5 Conclusions

Our study has demonstrated that the phonemic handshape inventory of CTSL, a village sign language, and NSL, a community sign language, are different in two important ways. CTSL’s handshape inventory has changed more slowly than NSL’s across the same time period, and, while the sizes of the two languages’ inventories are not different, handshape complexity is higher in NSL than in CTSL. In a community sign language, the continuous influx of new members (e.g. in a school setting) fosters a situation for rapid change and reorganisation. In other words, CTSL may be more like a slow cooker, and NSL more like a pressure cooker.

We have also introduced a new methodological diagnostic for including a feature in an inventory that is based not on minimal pairs, but rather on a dimension of productivity. This method allows near-minimal pairs to introduce a new feature into the system.

In stepping back and generalising these results to similar work on spoken language, we see that many factors guide the creation of phonology. A few of the parallels between signed and spoken languages are as follows. First, since we found that CTSL and NSL do not have inventories of different sizes, but their inventories differ in complexity, our findings suggest generally that inventory size and complexity should be investigated as distinct dimensions of phonological creation. Second, we found that the effect of community size is manifested as a positive link – the larger the community, the more complexity, while the effect of type of contact is an inverse relationship. When there is a community of signers, or (presumably) speakers, the complexity of the inventory is expected to decrease, perhaps as a response to signers being both producers and perceivers of the system. A third generalisation is that an emerging phonological system, just like any phonological system, cannot be treated monolithically. Different features or classes of features are subject to different types of biases and pressures.

This work has demonstrated that the phonological systems of handshape in emerging sign languages can be informative at many levels, and that factors observable only from a great distance in time and space in spoken languages are magnified in data from emerging sign languages.

## Supplementary Material

Brentari 2021 Supplementary

## Figures and Tables

**Table I:** The study groups categorised by type of contact.

	definition	horizontal contact	vertical contact
homesign	self-styled systems; little or no contact with other signers or homesigners	no	no
cohort 1: NSL1, CTSL1	contact with members of their own cohort	yes	no
cohorts 2/3: NSL2, CTSL2, CTSL3	contact with proficient signers from the previous cohort and from their own cohort	yes	yes

## Footnotes

1 De Vos & Pfau (2015) refer to the community/village distinction as an urban/rural one.

2 The handshapes in Fig. 2 are examples of the features in a ‘carrier’ handshape and thus stand for *groups* of handshapes. Moreover, the forms in Fig. 2 do not exhaust all possibilities for joint configuration and selected fingers, but represent the range of forms produced by signers in this study. Not all possible combinations of features are attested; for example, three-finger, ulnar handshapes (a high-complexity selected finger group) are not attested with all of the medium-complexity joint configuration features.

3 There are exceptions to this claim as well. In remote Polynesia there are very tight social networks with few external contacts and large amounts of communally shared information; these communities have small phoneme inventories (Trudgill 2011).

4 In fact, since school is typically where signers are first exposed to NSL, practically all NSL signers start out as homesigners.

5 Labels are typically nouns and events are typically verbs, but in the absence of grammatical diagnostics to confirm this, we have used the more neutral terms.

6 The active or ‘dominant’ hand can shift from one hand to another; it does so in Fig. 4b in the homesign example. Also note that the thumb is not taken into consideration in our notation system, since it displays so much variation in sign language phonology generally.

7 The complexity scores were treated as continuous interval variables, which are ordinal variables such as those of a Likert scale, since it is not clear that the difference between levels 1 and 2 is the same as the difference between levels 2 and 3. Complexity scores are restricted to the interval [1, 4], since 4 is the maximum handshape complexity value in the current dataset.

8 Available as online supplementary materials at https://doi.org/10.1017/S0952675721000336.

9 The R formula of the model is as follows: [DV] ~ 1 + Vertical + Horizontal + logCommunitySize + HS class + HS class : Vertical + HS class : Horizontal + HS class : logCommunitySize + (1 + HS class∣Participant) + (1 + Vertical + Horizontal + logCommunitySize + HS class + HS class ∶ Vertical + HS class : Horizontal + HS class ∶ logCommunitySize)∣∣Item) + (1∣Gesture), where [DV] is one of three complexity measures. We collected a total of 28,000 posterior samples from four chains (7000 samples per chain) using the No U-Turn Sampler implemented in Stan within the *brms* package. For every model parameter, all four chains of posterior samples were well mixed and converged (R_hat = 1). We then aggregated the posterior samples across chains for further analysis.

10 This may be true for spoken languages as well, since phonetic considerations are often used as diagnostic evidence for deciding which features are default and which features should be grouped together.
