# Supplementary material for: Community interactions and phonemic inventories in emerging sign languages
Source: Phonology. Author manuscript; Available in PMC 2025 Aug 16. (PMC12356508; doi:10.1017/S0952675721000336)
Supplement: Brentari 2021 Supplementary [file NIHMS2100132-supplement-Brentari_2021_Supplementary.pdf]

Phonology **38** (2021). Supplementary materials

Community interactions and phonemic inventories in emerging sign languages

Diane Brentari
University of Chicago
Rabia Ergin
Tufts University
Ann Senghas
Barnard College
Pyeong Whan Cho
University of Michigan
Eli Owens
University of Chicago
Marie Coppola
University of Connecticut

Supplementary materials

Appendix

Data by individual for average joint complexity, selected finger complexity and total complexity (the average of joint and selected finger complexity), as well as the average number of handshape types (distinct handshapes) and handshape tokens (uses of handshape).

group	average joint complexity	average selected fingers complexity	average total complexity	average types	average tokens
**CTSL1**	**1.87**	**1.21**	**1.54**	**36**	**179**
signer 1	1.98	1.12	1.55	37	191
signer 2	1.78	1.24	1.51	45	213
signer 3	1.80	1.22	1.51	30	125
signer 4	1.91	1.23	1.57	33	187
**CTSL2**	**1.74**	**1.15**	**1.44**	**37**	**197**
signer 1	1.65	1.22	1.43	40	223
signer 2	1.61	1.07	1.34	32	182
signer 3	1.87	1.16	1.52	42	200
signer 4	1.87	1.12	1.49	35	178
**CTSL3**	**1.82**	**1.23**	**1.53**	**37**	**192**
signer 1	1.87	1.15	1.51	37	194
signer 2	1.89	1.36	1.63	35	192
signer 3	1.82	1.23	1.52	39	177
signer 4	1.73	1.19	1.46	37	206
**Homesign**	**1.89**	**1.40**	**1.65**	**54**	**285**
signer 1	1.90	1.57	1.73	66	402
signer 2	1.90	1.24	1.57	47	278
signer 3	1.83	1.38	1.61	63	210
signer 4	1.94	1.27	1.61	40	251
**NSL1**	**1.78**	**1.43**	**1.60**	**31**	**186**
signer 1	1.88	1.44	1.66	33	201
signer 2	1.85	1.38	1.62	32	197
signer 3	1.75	1.41	1.76	30	145
signer 4	1.65	1.38	1.52	30	199
**NSL2**	**1.92**	**1.32**	**1.62**	**36**	**233**
signer 1	1.85	1.12	1.48	31	178
signer 2	1.82	1.23	1.52	30	257
signer 3	2.07	1.39	1.73	40	217
signer 4	1.94	1.22	1.58	41	315
signer 5	1.84	1.53	1.69	36	200
